# Molecular Epidemiology of HCV Monoinfection and HIV/HCV Coinfection in Injection Drug Users in Liuzhou, Southern China

**DOI:** 10.1371/journal.pone.0003608

**Published:** 2008-10-31

**Authors:** Yi Tan, Qi Hou Wei, Liu Jun Chen, Pui Chung Chan, Wen Sheng Lai, Ming Liang He, Hsiang Fu Kung, Shui Shan Lee

**Affiliations:** 1 Stanley Ho Centre for Emerging Infectious Diseases, The Chinese University of Hong Kong, Hong Kong, China; 2 Liuzhou Center for Disease Control and Prevention, Guangxi, China; 3 Department of Microbiology, The Chinese University of Hong Kong, Hong Kong, China; National AIDS Research Institute, India

## Abstract

**Background:**

Hepatitis C virus (HCV) mono-infection and HCV/HIV (human immunodeficiency virus) co-infection are growing problems in injection drug users (IDU). Their prevalence and genotypic patterns vary with geographic locations. Access to harm reduction measures is opening up opportunities for improving the HIV/HCV profiling of IDU in China, where IDUs account for a significant proportion of the two infections especially in the southern part of the country.

**Methodology/Principal Findings:**

A cross sectional study was conducted. Through the Liuzhou Methadone Clinic, a total of 117 injection drug users (IDUs) were recruited from Guangxi, Southern China. A majority of the IDUs (96%) were HCV antibody positive, of which 21% were HIV infected. Unlike HCV monoinfection, there was spatial heterogeneity in the distribution of HIV/HCV coinfection, the latter also characterized by a higher prevalence of needle-sharing. Phylogenetic analysis revealed that genotype 6a was predominant in the study population. There were shorter genetic distances among the 6a sequences compared to the other HCV subtypes-1a, 3a, and 3b.

**Conclusion/Significance:**

The results suggested that HIV and HCV were introduced at around the same time to the IDU populations in Southern China, followed by their differential spread as determined by the biologic characteristics of the virus and the intensity of behavioural risk. This pattern is different from that in other South East Asian countries where HCV infections have probably predated HIV.

## Introduction

Hepatitis C Virus (HCV) infection is a worldwide health problem characterized by the lack of an effective vaccine, need for expensive treatment, chronicity of morbidity and associated mortality [1]. HCV shares the same transmission routes with human immunodeficiency virus (HIV). It is however more efficiently transmitted through exposure to contaminated blood, as well as needle-sharing in injection drug users (IDUs). Worldwide, more than 170 million people are infected by HCV, more than 4 times the number of people living with HIV. The prevalence of HIV/HCV coinfection in IDUs is also high, ranging from 50% to less than 10% in different regions [2], [3]. HCV infection has become one of the most important causes of chronic hepatitis, cirrhosis, and hepatocellular carcinoma in China. Currently, more than 70% of IDUs in Southern China are infected with HCV [4]. Most have been reported in Yunnan, Guangxi, and Guangdong province [4], [5].

HCV is a spherical, enveloped virus with a linear positive sense RNA genome of approximately 9.6 kb in length [6]. One of the most important features of HCV is its high degree of genetic variability. Variations of the HCV genome have led to its classification into six major genotypes and a large number of subtypes. The different genotypes display up to 70% sequence similarity, whereas subtypes vary by more than 20% [7]. The open reading frame (ORF) encodes the structural proteins core (C), envelope (E1, E2), and the non-structural proteins (NS2, NS3, NS4a NS4b, NS5a and NS5b) [6]. Region E2 displays most sequence diversity, whereas 5_UTR and CORE sequences are more conserved [8], [9]. Response to interferon (IFN)-based therapies in patients infected with HCV genotype 4 and 1 is much lower than that for genotypes 2 and 3 [10]. Clinically, genotyping of HCV is therefore important for predicting treatment responses and for determining the duration of antiviral therapy. HCV genotyping is also of public health importance as it can be useful for investigating outbreaks and for understanding the epidemiology of the infection [5], [11]. To date, genotypes 1, 2 and 3 have a worldwide distribution while the others are more localized. Genotype 4, for example, is mostly found in Middle East and North Africa; while 5 is common in South Africa and 6 in South East Asia [12]. So far most studies have focused on HCV genotype 1, 2, and 3 because they are pandemic, but relatively little is known about the epidemiology of genotype 6 which is circulating in South East Asian countries and neighboring China.

Southern China borders the South East Asian countries of Myanmar, Laos, and Vietnam, the latter forming the Golden Triangle, one main producer of heroin in the region. Guangxi is the neighbor of Yunnan province, which is becoming an important focal point for trafficking drugs from countries of South East Asia to inner China [4]. In anticipation of the rising threat of HIV/HCV infections, methadone treatment programme was initiated in China [15]. Methadone clinics provide access of harm reduction measures to high risk populations. We undertook to examine the molecular epidemiology of HIV and HCV infections through these new outlets.

## Materials and Methods

### Study participants

Drug users were recruited from the Liuzhou Methadone Clinic in Guangxi, Southern China, from August 2006 to October 2006. The clinic is run by Liuzhou Center for Disease Control and Prevention (Liuzhou CDC). Written consent was obtained and the participants were interviewed by trained research staff to determine their demographics, drug addiction patterns, and sexual risk behaviours. Blood samples were centrifuged, aliquoted and stored at −70°C before testing for HIV and HCV antibody, and for HCV genotyping. Patients were informed that the tests requested in the study were designed for epidemiological research. As regards clinical testing, all patients at the methadone clinics had access to HIV tests and related counseling by professional staff. Those tested positive were referred to the specialist clinic run by the same CDC, where standard antiretroviral therapy was prescribed in accordance with national treatment guidelines. HCV counseling and treatment were however not available as a standard practice, and this study served to determine the dimension and characteristics of the condition. In our study protocol, communication of laboratory test (HIV and HCV) results to recruited clients was made on request. Ethical Approval, covering the study protocol, was obtained from Ethics Committee of the Chinese University of Hong Kong.

### Serological assays

HIV and HCV antibody tests were performed at the Liuzhou CDC. HIV antibody status was determined by Anti-HIV 1+2 Antibody ELISA Kit (Double Antigen Sandwich). Positive samples were confirmed by Western blot assay. The presence of HCV antibody was determined by ELISA.

### HCV RNA extraction, RT-PCR amplification, and sequencing

HCV RNA was extracted from 200 µL of plasma by using PureLink™ Viral RNA/DNA Mini Kit (Invitrogen), followed by standard protocol. cDNA was synthesized from 5 µL extracted RNA with SuperScript III First-Strand Synthesis System (Invitrogen). The primers of NS5B region for nested PCR are described elsewhere [16] with slight modification: Forward (OF) 5′- TAT GAC ACC MGY TGC TTT GAY TC-3′, Outreverse (OR) 5′- TTG GAG GAG CAD GAT GTT ATS AGC TC -3′, Innerreverse (IR) 5′- GAR TAC CTG GTC ATA GCC TCC GT -3′. First round PCR using primer OF and OR was conducted with the following conditions: 95°C for 5 min, then 30 cycles of 95°C for 30 sec, 56°C for 30 sec and 72°C for 40 sec. Second round PCR using primer OF and IR was conducted with the same condition except that the annealing temperature is 52°C. The forward primer OF was used to sequence the PCR product.

### HCV genotyping and phylogenetic analyses

HCV genotype was determined after alignment with reference sequences from the HCV database (available at: http://hcv.lanl.gov/content/hcv-db/BASIC_BLAST/basic_blast.html) followed by phylogenetic analysis. Sequences were aligned using CLUSTAL_X and then edited by BioEdit. Phylogenetic analysis was performed with MEGA 3.0. The neighbor-joining method was used with 1000 bootstrap replications (Kimura 2-parameter Substitution Model). The reference sequences used in phylogenetic analysis were obtained from the Genebank: D17763 (3a, Japan), X76918 (3a, Germany), D28917 (3a, Japan), D49374 (3b, Japan), AY460204 (1b, China-Shanghai), M84754 (1b, Taiwan), AJ238800 (1b, Germany), NC_004102 (1a, USA), M62321 (1a, USA), D10749 (1a, Japan), AY973866 (6a, Hong Kong), AY859526 (6a, Hong Kong), Y12083 (6a, Hong Kong), D84262 (6b, Thailand), AY878650 (6k, China-Kunming), D84264 (6k, Vietnam), D84265 (6h, Vietnam), D63822 (6g, Indonesia), and D84263 (6d, Vietnam). The sequences of subtype 6a in other countries or regions for phylogenetic analysis were randomly selected from Genbank: AY834940 (China), AY834941 (China), AY834942 (China), AY834943 (China), AB204705 (Hong Kong), AB204706 (Hong Kong), AB204707 (Hong Kong), AB204708 (Hong Kong), D17490 (Vietnam), D155502 (Vietnam), D155503 (Vietnam), D17475 (Taiwan), D17474 (Taiwan), D17488 (Taiwan), L38379 (Vietnam), DQ666268 (Taiwan), DQ663599 (Taiwan), DQ666264 (Taiwan), DQ663598 (Taiwan), DQ663600 (Taiwan).

### Statistical analysis

SPSS 13.0 was used to analyze the data. Univariate analysis was performed with Chi-square or Fisher's exact test for each risk factor. Multivariate logistic regression model was used for multivariate analysis. Factors giving a *p* value of less than 0.1 in the univariate analysis were included in the logistic regression model. A *p* value of less than 0.05 was considered significant.

## Results

### Prevalence of HIV infection, HCV infection, and coinfection among IDUs in Liuzhou, Guangxi, Southern China

A total of 117 IDUs were recruited from the Liuzhou Methadone Clinic during the two-month study period. Of these 112 subjects were HCV infected as determined by the antibody test, giving a prevalence of 96%. Among the HCV positive IDUs, 25 (21%) had HIV/HCV coinfection, while the remaining 87 were HCV monoinfection. None of the subjects was HIV positive but HCV negative.

### Demography and risk behavior pattern

Table 1 shows the demography, risk factors, and the pattern of methadone use in HIV/HCV coinfection and HCV monoinfection. In univariate analysis, there were no significant differences between coinfection and monoinfection in the respondents' age, gender, education level, smoking status, drinking status, and previous rehabilitation history. The geographic distribution of infections differed, with Yufeng and Chengzhong district having more HIV/HCV coinfections, whereas HCV was evenly distributed in the 4 districts of the city. Most HIV/HCV coinfected subjects were unemployed, while only half of the HCV monoinfected IDUs were unemployed (76% vs. 54%, p<0.05). More HIV infected subjects had been in prison (76% vs. 56%, χ^2^ = 6.753, p<0.01). As regards injection behaviors, there was significantly higher prevalence of needle sharing in coinfections (73% vs. 36%, χ2 = 12.128, p<0.01). First injection before 1995 was also associated with coinfection. There was however no significant difference between the two groups in condom use or multiple sex partners. A stable daily methadone dose of over 40 mg was associated with HIV infection (64% vs. 39%, χ^2^ = 4.88, p<0.05).

**Table 1:** Demographics, risk behaviors, and methadone use patterns of HIV/HCV coinfected and HCV monoinfected subjects in the study population (n = 112).

Factor	HIV/HCV coinfection (n = 25)	HCV monoinfection (n = 87)	χ^2^	df	*p* (Chi-square)
**Demography**					
Birth on 1965 or after	21	70	–	–	0.780^a^
Male gender	17	67	0.841	1	0.359
District of residence					
Liubei	2	18			
Liunan	3	32	13.508	3	0.009
Yufeng	15	33			
Chengzhong	5	4			
Educational attainment at high school or above	5	32	2.472	1	0.116
Unemployed	19	47	3.875	1	0.049
Smoking status	24	86	–	–	0.389^a^
Drinking status	2	16	–	–	0.354^a^
Previous rehabilitation	22	72	–	–	0.759^a^
Previous imprisonment	19/22*	49	6.753	1	0.009
**Drug abuse risk behaviors**					
Initiation of drug use before 1995	17	40	3.769	1	0.070
Initiation of injection before 1995	10/20*	13/67*	7.414	1	0.006
History of needle sharing	19/25	29/80	12.128	1	*p*<0.001
Injection in the past 1 month	9/25	24/80	0.318	1	0.573
Needle sharing in the past 1 month	3/9	6/24	–	–	0.677^a^
**Sexual risk behaviors**					
Non regular partner	7	21	0.154	1	0.694
Condom use in last 3 months	3/7	13/21	–	–	0.418^a^
**Methadone use pattern**					
Defaulted for ≤3 days in the last 1 month	22	74	–	–	1.000^a^
Stable methadone dose >40 mg	16	34	4.880	1	0.027

* Missing data have been excluded.

a Fisher's exact test.

Multivariate analysis was performed by logistic regression. Factors that gave a *p* value of less than 0.1 in univariate analysis were included in the logistic regression model. These were: district of residence, employment status, previous imprisonment, initiating drug use before 1995, first injection before 1995, needle sharing, and stable methadone dose >40 mg. District of residence and needle sharing were independently associated with HIV/HCV coinfection in the regression model (table 2).

**Table 2:** Multivariate logistic regression analysis for risk factors associated with HIV/HCV coinfection.

	B	SE	*P* value	Odds ratio	95% CI
**Residential district of Chengzhong**	2.973	1.392	0.033	19.550	1.277–299.314
**Needle sharing**	2.164	0.753	0.004	8.708	1.989–38.116

### HCV genotyping

**Figure 1:**
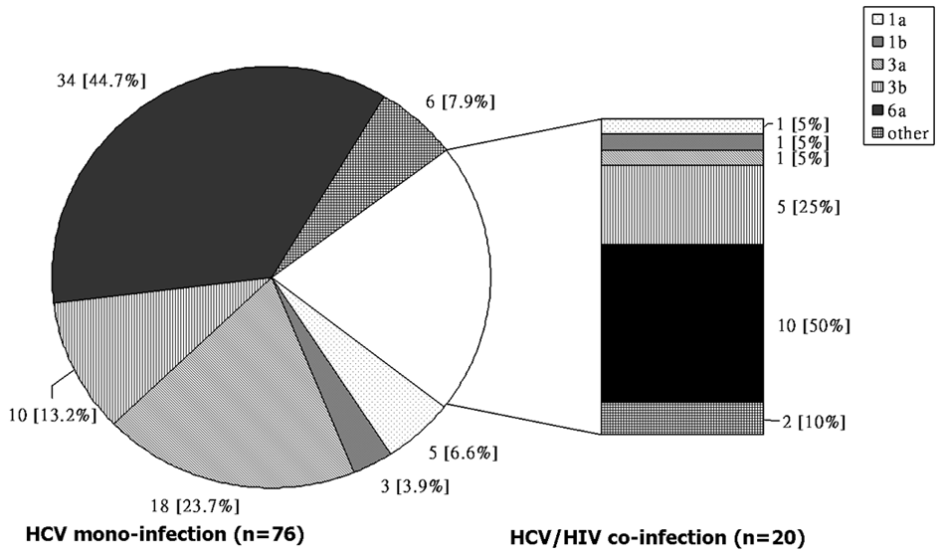
HCV genotypes, by HIV status, among 96 drug users in Liuzhou cohort.

Ninety-six of the 112 HCV infected subjects were RNA positive (86%). Among them, 20 were HIV/HCV coinfected, and 76 were HCV monoinfected. Overall, subtype 6a (46%) was predominant, followed by subtype 3a (20%) and 3b (16%). Figure 1 displays the genotype distribution by HIV status. There was no significant difference in HCV genotype distribution between HIV/HCV coinfection and HCV monoinfection (χ^2^ = 5.149, p = 0.356). There were more HCV genotype 3a in HCV monoinfection than in coinfection patients (Figure 1).

### Phylogenetic analysis

**Figure 2:**
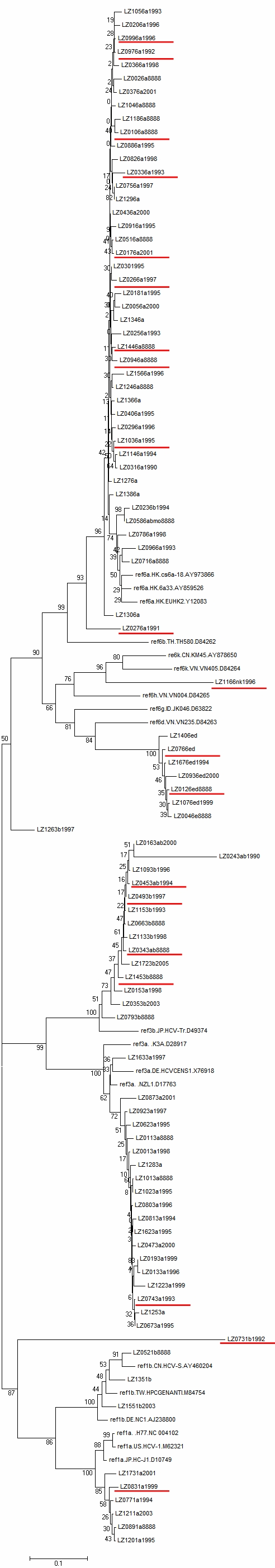
HCV NS5B phylogenetic tree for drug users in Liuzhou, Guangxi, Southern China, with 96 drug users of which 20 are HIV/HCV co-infected (underlined).

**Figure 3:**
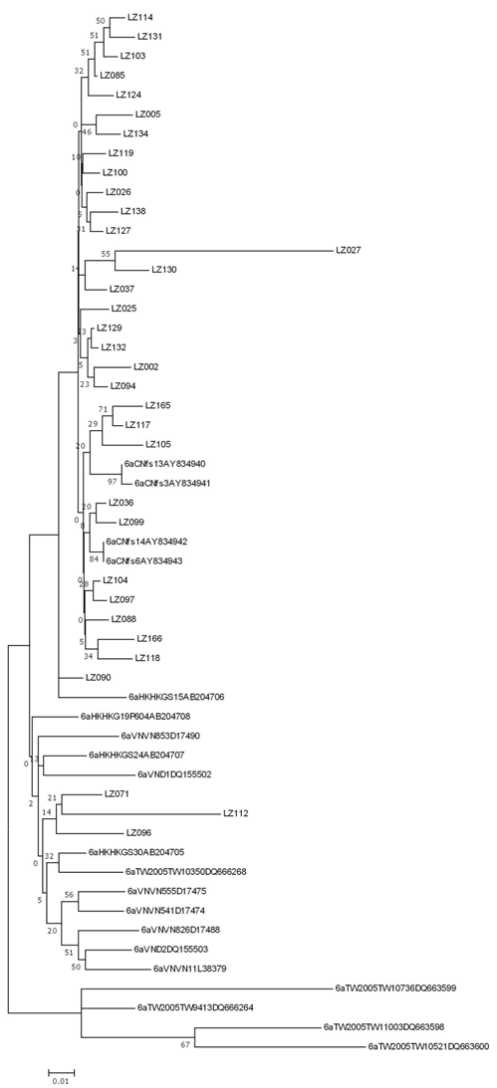
HCV genotype 6a in the study population (n = 34) and other countries and regions. Codes for sources of samples: CN = China; TW = Taiwan; HK = Hong Kong VN = Vietnam.

Figure 2 shows the phylogenetic tree of 96 sequences and reference sequences. The sequences of coinfection IDUs were evenly distributed in the whole tree. There is no evidence of clustering in HIV coinfected patients. Comparing between different genotypes, the genetic distances obtained from MEGA 3.0 within genotype 6 (0.125±0.012) were shorter than those within genotype 1 (0.237±0.021) and genotype 3 (0.168±0.017). The 6a sequences from Liuhzou were aligned with other 6a sequences randomly selected from the Genebank. Overall, there's a high degree of sequence identity among the 6a samples in Liuzhou, than between sequences in this study and those in neighbouring cities/countries in Hong Kong, Tauiwan, Vietnam and China (Figure 3).

## Discussion

In our study, almost all IDUs in Liuzhou, Guangxi, were HCV infected. About one fifth were HIV/HCV coinfected, and which appeared to be localized in one of the four districts (Yufeng district) of the city. The predominant HCV genotype was 6a, the sequence of which showed a high degree of identity, followed by 1a, 3a, and 3b, which were circulating in smaller and relatively equal proportions. Compared with HCV monoinfection, HIV/HCV coinfection was associated with a higher prevalence of risk behaviours. We postulate that both HIV and HCV infections were introduced to the IDU population in Southern China almost simultaneously, probably in the 1990s. The specific patterns of the two infections can be explained by the geography and history of heroin addiction in Liuzhou.

Located at the crossroad of Guangxi, Liuzhou is linking itself with cities of Yunnan, Guizhou, Hunan, and Guangdong provinces on one hand, and bordering Vietnam on the other. As a hub of the drug trafficking route from the Golden Triangle, the problem of addiction began to escalate in Liuzhou since the late 1980s, when HIV was reported in IDUs in the neighbouring countries. The predominance of genotype 6a (46%) is consistent with results in other reports on IDUs in Southern China [4]. Some HCV genotypes have a very restricted geographic distribution. Genotype 6 is one such example which appears to be localised in South East Asia especially Vietnam and Thailand. Unlike Northern China where genotype 1 (especially 1b) and 3 predominate [5], [17], Southern China is characterized by the high prevalence of genotype 6 [4], [5]. There are two possible interpretations. The first is that this is part and parcel of the rapidly expanding epidemic of genotype 6a from South East Asia, which then overspills into Guangxi. The second is that a longstanding endemic of HCV type 6 in South East Asia including Southern China has predated the HCV pandemic. The trafficking of heroin alongside the practice of unsafe injection behaviours have resulted in the explosive spread of genotype 6a in the local drug taking community. The second interpretation could explain the co-existence of a multitude of subtypes of genotype 6 characterized by extensive sequence variability [7], [18], which have probably resulted from a long evolutionary process over time. A similar endemic spread seems to have occurred in the Indian subcontinent with genotype 3, in Western Africa with genotype 2, and in Egypt with genotype 4 [19], [20], [21]. In contrast, the relatively low proportion of other HCV genotypes may reflect their introduction in Southern China from other areas, possibly Northern China, Japan or Europe. The identity of the 6a sequences in Liuzhou suggested that the virus was introduced almost simultaneously over a short period of time in the IDU population.

The relatively lower HIV prevalence in the same IDU population needs to be accounted for. Biologically, HIV transmission is less efficient than HCV infection. This is perhaps the most important reason why the prevalence of HIV in Liuzhou was only one-fifth that of HCV infection. The localization of HIV/HCV coinfection in some but not all of the districts in Liuzhou suggested that transmission has been less efficient and more strongly associated with risk-taking behaviours, as illustrated in our findings. Interestingly, previous imprisonment was also found to be associated with HIV/HCV coinfection. Imprisonment may simply be a surrogate of a higher demand for heroin, and therefore a marker for higher prevalence of risky behaviours. Outbreak of HIV infection in prison resulting from needle sharing is another possibility though this cannot be proven in our study. We also tested the association between HIV infection and the timing for initiating drug injection. IDUs who started heroin use or initiated injection before 1995 were more likely to have contracted HIV infection. The year 1995 was chosen as the cutoff because it was the same year that the national government began to crack down on heroin addiction as a means to control HIV spread. This was theoretically also the same timepoint that the association between HIV transmission and injection drug use was officially acknowledged. The results may mean that IDUs after 1995 were more aware of the risk of HIV and could have reduced the level of risk, which is in line with a health belief model. Alternatively, this may also be a reflection of the phenomenon that longer duration (thus before 1995) of addiction was associated with enhanced risk of exposure. From a molecular perspective, the HCV sequences of coinfection group did not reveal any clustering, suggesting that HIV could have been introduced at the same time with HCV in Liuzhou after 1990s.

Knowingly our research carries some limitations especially that on sampling. Our subjects were recruited exclusively from the city's first methadone clinic. It may be argued that methadone clients were highly selected, and that their profile of risk behaviours, and their geographic distribution may have been biased. It's possible that IDUs who are still actively injecting heroin could be missed, thus underestimating the risk levels in the analysis. On the other hand, heroin users who have been injecting drugs for a long period of time were preferentially recruited by methadone clinics, so older IDUs could have been over-represented. As a hard-to-reach population, there is no perfect sampling frame for IDUs in China. The HCV and HIV results are robust enough to reflect the situation in a significant proportion of IDUs in Southern China. Another limitation is that the analysis was based on results on self-reported behaviours. Though the questionnaires were administered by trained research staff, reliability of such results would be difficult to be ensured. Extrapolation of results in this study to the entire IDUs populations in Southern China should be considered with care.

In conclusion, our study suggested a short history of the introduction of both HIV and HCV infections to IDUs in Liuzhou, Southern China. This is different from Northern China where HCV genotype 1b predominated largely because of the use of contaminated blood products before 1990s. This was followed by HIV spread through the same transmission route [5]. As exemplified in our study in Liuzhou, HCV and HIV infections took root in Southern China after 1990s through needle sharing in IDUs. In the neighbouring city of Hong Kong, the situation was different despite its similar location in Southern China. Characteristically, methadone clinics were established in Hong Kong in the 1970s, long before HIV spread in any IDU population in the world was reported. Todate HCV infection had affected a majority of the IDUs in Hong Kong but HIV/HCV coinfection was distinctly uncommon. [22]. Interestingly Genotype 6a is also common in Hong Kong, which may again reflect the transmission of an endemic strain before the HIV pandemic. In other countries of South East Asia, such as Thailand, Myanmar in the Golden Triangle HCV might have spread first through the drug trafficking routes that have been in existence for decades. The spread of HIV took place more recently only when the virus landed on the IDUs, as manifested in the epidemiologic pattern of Thailand [23]. The extremely high HCV prevalence (96%) in IDUs in Liuzhou is a cause for concern. There is the risk that the virus may spread from IDUs to the general public, and then propagated through unprotected sex alongside needle-sharing. The high HIV prevalence in IDU also means that with time, a significant number of IDUs would develop complications arising from chronic HCV infection. Prevention, diagnosis and treatment of HIV/HCV co-infection would become another clinical cum public health challenge in Guangxi, Southern China.
